# Efficacy of repetitive transcranial magnetic stimulation for consciousness recovery in children with disorders of consciousness following traumatic brain injury

**DOI:** 10.3389/fneur.2025.1678379

**Published:** 2025-10-13

**Authors:** Nan Zheng, Zhuo Zou, Wenjuan Wang, Fangling Dong, Xuemei He, Jian Ren, Xiaoyan Liu, Yangping Zhang, Zhongjian Su

**Affiliations:** ^1^Department of Rehabilitation, Kunming Children’s Hospital, Kunming Medical University, Kunming, Yunnan, China; ^2^Department of Pediatric Intensive Care Unit, Kunming Children’s Hospital, Kunming Medical University, Kunming, Yunnan, China; ^3^Department of Cardiology, Kunming Children’s Hospital, Kunming Medical University, Kunming, Yunnan, China

**Keywords:** repetitive transcranial magnetic stimulation (rTMS), imperiods of consciousness, traumatic brain injury (TBI), pediatrics, high-frequency repetitive transcranial magnetic stimulation, dorsolateral prefrontal cortex, restored consciousness, neurorehabilitation

## Abstract

**Objective:**

To evaluate the efficacy of 5 Hz repetitive transcranial magnetic stimulation (rTMS) over the left dorsolateral prefrontal cortex (left DLPFC) for consciousness recovery in children with disorders of consciousness (DOC) following traumatic brain injury (TBI).

**Methods:**

This randomized controlled trial included 98 pediatric patients aged ≥2 years with DOC after TBI, admitted to Kunming Children’s Hospital from January 2023 to July 2025. Patients were randomly divided into an experimental group (*n* = 49) and a control group (*n* = 49). The experimental group received 5 Hz rTMS targeting the left DLPFC (80% resting motor threshold, 1,000 pulses per session, totaling 20 min), combined with conventional rehabilitation therapy (once daily for 3 weeks). The control group received only conventional rehabilitation therapy. The primary efficacy outcomes included serum neuron-specific enolase (NSE) level, Coma Recovery Scale-Revised (CRS-R) score, Glasgow Coma Scale (GCS) score, and level of consciousness before and after treatment.

**Results:**

After 3 weeks of intervention, the experimental group demonstrated statistically significant improvements compared to both baseline status and the control group (*p* < 0.05). The experimental group demonstrated a significant reduction in serum NSE levels, a significant increase in CRS-R and GCS scores, and a significant improvement in the level of consciousness. No adverse events (including seizures) were observed throughout the treatment.

**Conclusion:**

This study provided the first clinical evidence that the combined application of 5 Hz rTMS targeting the left DLPFC is a safe and effective intervention for promoting the recovery of consciousness in children with DOC following TBI. Significant improvements in behavioral scales (CRS-R, GCS) and reduced levels of the neurological injury marker (serum NSE) suggest that this protocol exerts dual effects of promoting arousal and neuroprotection. This novel treatment approach, designed based on the characteristics of pediatric neurological development, offers a promising non-invasive neuromodulation strategy for this challenging patient population, filling a critical evidence gap in this field.

## 1 Introduction

TBI is an acute head injury caused by direct or indirect external violence to the head, which can lead to DOC and represents a significant public health issue (1). The global incidence of pediatric TBI ranges from 47 to 280 cases per 100,000 people annually, affecting over 3 million children worldwide each year (2). Some researchers report that the incidence of TBI in Chinese children is between 55.4 and 64.1 cases per 100,000 people, with a male-to-female ratio of 2.98:1 (3). Retrospective studies on pediatric TBI patients indicate that approximately 54% of children admitted in Unresponsive Wakefulness Syndrome (UWS) or minimally conscious state (MCS) regain consciousness within one year, while 46% remain in a prolonged DOC (4). The large number of TBI patients experiencing DOC, coupled with slow recovery rates, imposes a substantial financial burden on healthcare systems and significantly reduces the quality of life for patients and their families.

With population growth and inadequacies in construction, road infrastructure, and sports safety measures, TBI incidence has risen rapidly. Falls from heights, traffic accidents, and child abuse are primary etiologies of TBI (5, 6). Unlike adults, children possess distinct anatomical features including relatively larger head size, lower cerebral blood volume, thin cranial bones, high cerebral water content, reduced myelination, incompletely aerated paranasal sinuses, and limited cerebrospinal fluid (CSF) cushioning. These factors collectively increase TBI susceptibility (5). Currently, the diagnosis and management of pediatric TBI primarily aim to reduce mortality, focusing on life-saving interventions for critical and severe cases. Although the concept of critical rehabilitation has gained widespread acceptance, efforts remain centered on protecting musculoskeletal and cardiopulmonary functions. Notably, there is still a lack of clearly effective management strategies and clinical guidelines for pediatric patients with DOC.

Transcranial Magnetic Stimulation (TMS) is a non-invasive neuromodulation technique employed in the management of neurological disorders and the investigation of brain function. High-frequency rTMS (frequency >1 Hz) enhances cortical excitability, while low-frequency rTMS (frequency ≤1 Hz) tends to suppress neural activity. A review of recent domestic and international literature indicates that multiple systematic reviews and meta-analyses have validated the efficacy of high-frequency rTMS in improving consciousness levels among adult patients (4, 7–12). Across these studies, the targeted brain regions and stimulation frequencies varied, with the left DLPFC being the most frequently targeted, followed by the primary motor cortex (M1) and the posterior parietal cortex (PPC). Preferred stimulation frequencies primarily included 10 Hz or 20 Hz, and one Chinese study also demonstrated significant therapeutic efficacy with 3 Hz stimulation (13). Notably, the incidence of adverse reactions associated with 20 Hz rTMS was significantly higher than that observed with 10 Hz rTMS (14).

NSE is a glycolytic enzyme predominantly localized in neurons and neuroendocrine cells. It is present at extremely high concentrations in the brain, while levels are relatively low in the peripheral nervous system and nearly undetectable in non-neural tissues (15–17). Following neuronal injury or death, NSE is released into the extracellular space, leading to elevated levels in both cerebrospinal fluid (CSF) and serum (16, 17). In the context of TBI, NSE concentrations have been shown to correlate with elevated intracranial pressure, severity of brain injury, and adverse clinical outcomes (15, 18, 19). Moreover, studies have demonstrated a significant negative correlation between serum NSE levels and GCS scores, supporting its role as a widely investigated biomarker for TBI (20).

To date, there are no direct clinical studies investigating rTMS application in pediatric patients with DOC following TBI. Children are by no means ‘small adults; their nervous systems remain incompletely developed, rendering them more sensitive to external stimuli after severe trauma. Consequently, the application of rTMS in this population requires careful consideration of neurodevelopmental stage, clinical characteristics, and safety profiles. Direct adaptation of adult rTMS protocols may precipitate adverse events such as epilepsy. Given these multifaceted factors, we proposed a novel stimulation protocol: 5 Hz high-frequency rTMS targeting the left DLPFC for consciousness recovery in pediatric patients with DOC following TBI. This approach aims to evaluate its potential to effectively improve conscious states in this vulnerable group.

## 2 Materials and methods

### 2.1 Participants

Ninety-eight pediatric patients with DOC following TBI admitted to Kunming Children’s Hospital from January 2023 to July 2025 were enrolled in this study (approved by the Institutional Review Board of our hospital, with written informed consent obtained from their legal guardians). Participants were randomly divided into a control group and an experimental group, with 49 cases in each group. No significant differences were observed in baseline characteristics between the two groups, including sex, age, etiology of TBI, serum NSE level (normal range: 0–15.7 ng/mL), GCS score, CRS-R score, and level of consciousness (all *p* > 0.05) as shown in Table 1.

**Table 1:** General characteristics of the experimental group vs. control group

Group	n	Sex		Etiology			Age(years) M(*P*_25_,*P*_75_)	NSE (ng/mL) M(*P*_25_,*P*_75_)	GCS score M(*P*_25_,*P*_75_)	CRS-R score M(*P*_25_,*P*_75_)	Level of consciousness	
		Boy	Girl	Traffic Accident Injury	Fall from Height Injury	Other external injuries					UWS	MCS
Experimental Group	49	39	10	27	15	7	6.25 (3.00,10.10)	138.16 (80.00,170.83)	9 (7,9)	8 (6,11)	16	33
Control Group	49	34	15	31	12	6	6.08 (4.00,7.73)	142.63 (32.33,168.98)	9 (7,9)	8 (5.5,10)	15	34
*χ^2^*/*z*		1.342		0.686			0.837	1.176	0.616	0.143	0.047	
*P*		0.247		0.710			0.403	0.240	0.538	0.886	0.828	

#### 2.1.1 Inclusion criteria

1. Meeting the diagnostic criteria for TBI and DOC (21, 22);
2. Age 2–18 years;
3. Disease duration < 3 months;
4. Generally stable clinical condition;
5. No prior formal rehabilitation training for improving disorders of consciousness before the initiation of intervention;
6. Written informed consent voluntarily provided by the legal guardian.

#### 2.1.2 Exclusion criteria

1. Presence of contraindications to rTMS treatment: (a) large-area skull defect; (b) history of epilepsy; (c) metallic implants (e.g., vascular stents, ventriculoperitoneal shunts, cranial repair materials, titanium cranial screws, external stimulators);
2. Critically ill patients with unstable vital signs in the acute phase;
3. Pre-existing motor or intellectual developmental disorders, or a history of brain injury prior to the current trauma;
4. Patients taking medications that affect cortical excitability.

### 2.2 Study design

The children in the experimental group received rTMS at a frequency of 5 Hz combined with conventional rehabilitation therapy, which included 30 min of passive range-of-motion exercises for the four limbs, 30 min of sensory stimulation therapy (encompassing music stimulation therapy, light stimulation therapy, gravity stimulation therapy, etc.), and acupuncture treatment, administered once daily for 3 consecutive weeks. The control group only underwent the same conventional rehabilitation therapy without receiving rTMS.

As a traditional Chinese therapeutic modality, acupuncture has demonstrated potential efficacy in the treatment of DOC in previous studies (23–27). The treatment protocol for this trial was designed based on previously published research (23–25, 28, 29). The primary acupoints selected were Baihui (GV20), Shuigou (GV26), Neiguan (PC6), and Zusanli (ST36). The procedure was performed as follows: The child was placed in a supine position. Following local skin disinfection, disposable sterile acupuncture needles (0.25 mm in diameter × 25 mm in length) were used. The insertion techniques were applied as follows: a subcutaneous insertion at Baihui (GV20), an upward oblique insertion at Shuigou (GV26), and perpendicular insertions at both Neiguan (PC6) and Zusanli (ST36). The needles were retained in place for 20 min.

rTMS Stimulation Protocol: The Magneuro60 TMS device (Nanjing Vishee Medical Technology Co., Ltd.) was used. With reference to the international 10–20 system for scalp electrode positioning, the stimulation site was defined as 1 cm anterior to the C3 electrode site. The minimum stimulation intensity required to elicit a motor evoked potential (MEP) with an amplitude (sinusoidal waveform) of at least 50 μV in the abductor pollicis brevis muscle point of the contralateral hand in at least 5 out of 10 consecutive stimuli was designated as the resting motor threshold (RMT). The child lay in a supine position. A figure-8 coil was used to deliver stimulation to the corresponding site of the left DLPFC (the F3 electrode site). The coil face was maintained in close contact with the scalp surface and remained tangent to the cranial surface. Key stimulation parameters were as follows: frequency of 5 Hz, intensity set at 80% of RMT, stimulation duration of 2 s, inter-stimulus interval of 10 s, total session duration of 20 min, 100 total stimulation cycles, and 1,000 total pulses.

### 2.3 Monitoring indicators

At baseline (post-enrollment and prior to intervention), all pediatric subjects were assessed with the CRS-R, GCS, and serum NSE level measurements. These evaluations were repeated following trial completion. Comparative analyses were performed both between groups and within groups. All assessments throughout the trial were performed by the same physician from the Assessment Division of the Department of Rehabilitation Medicine at Kunming Children’s Hospital. This physician was blinded to both the group allocation and the experimental protocol of the trial participants. Additionally, all serum NSE level tests used the same type of reagent from the same manufacturer.

### 2.4 Statistical analyses

SPSS 25.0 software was used for statistical processing and analysis. Count data were expressed as frequency (*n*) and percentage (%), and intergroup comparisons were conducted using chi-square test, corrected chi-square test, or Fisher’s exact probability method. Quantitative data that conformed to normal distribution and homogeneity of variance were expressed as mean ± standard deviation (x̅ ± s), and intergroup comparisons were performed using the t-test or analysis of variance; for data not conforming to normal distribution, they were expressed as M (P₂₅, P₇₅), and intergroup comparisons were conducted using the rank sum test. Statistical significance was set at *p* < 0.05.

## 3 Results

### 3.1 General condition of the patient

As shown in Table 1, among 98 pediatric patients with DOC following TBI, there were 58 cases (59.2%) due to traffic accidents, 27 cases (27.6%) due to falls from height, and 13 cases (13.2%) due to other external injuries. The cohort comprised 73 boys and 25 girls, with a male-to-female ratio of 2.9:1. No significant differences were observed between the two groups in baseline characteristics, including sex (*p* = 0.247), age (*p* = 0.403), etiology (*p* = 0.710), serum NSE level (*p* = 0.240), GCS score (*p* = 0.538), CRS-R score (*p* = 0.886), and level of consciousness (*p* = 0.828) (all *p* > 0.05).

### 3.2 Changes in monitoring indicators before and after treatment

**Figure 1:**
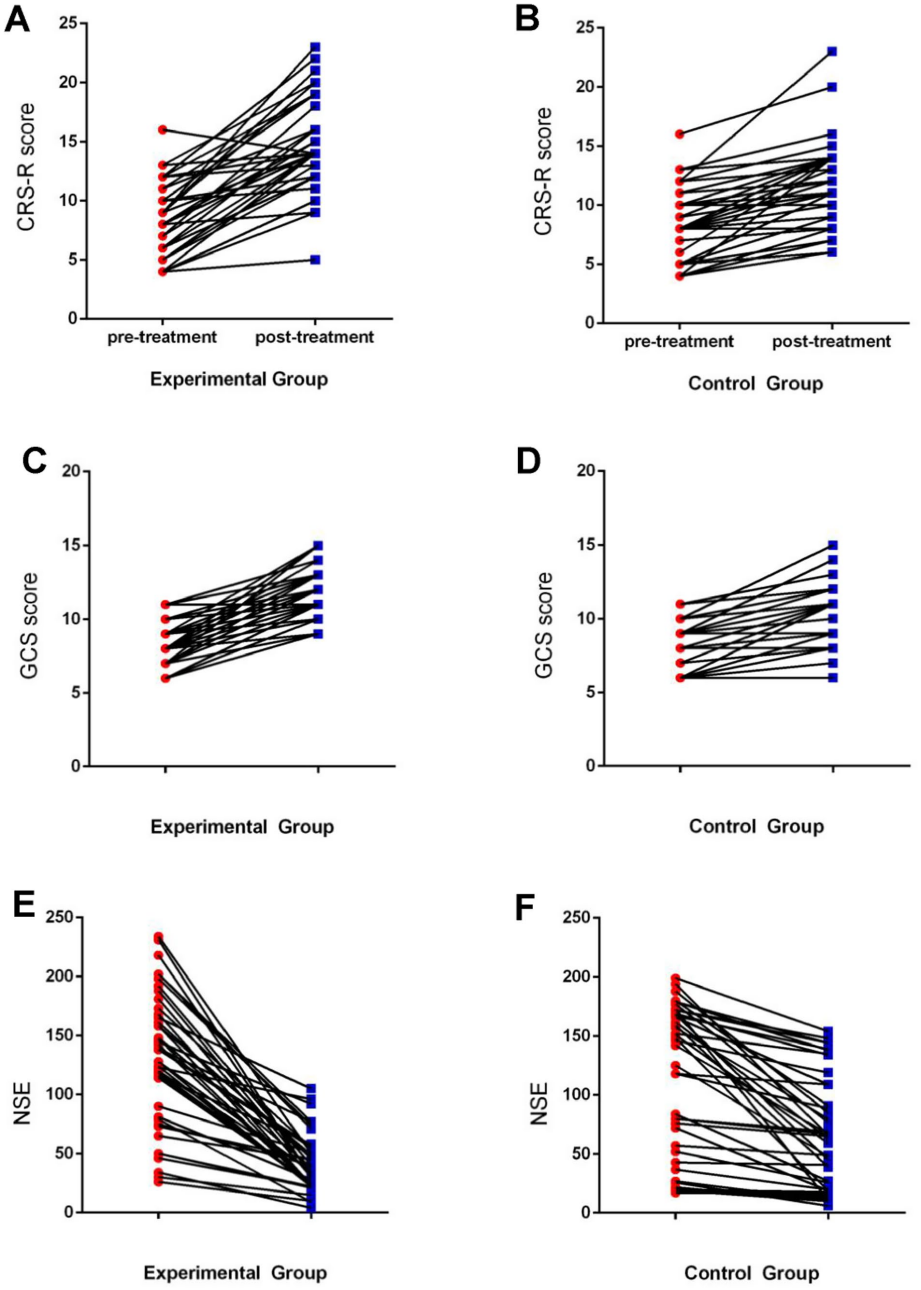
Changes in monitoring indicators before and after treatment.

As shown in Table 2 and Figure 1, serum NSE levels decreased significantly after treatment in both the experimental and control groups (both *p* < 0.001), with the experimental group showing significantly lower values than the control group (*p* = 0.047). Similarly, both groups demonstrated significant improvements in GCS scores after treatment (both *p* < 0.001), and the experimental group achieved significantly higher scores compared to the control group (*p* < 0.001). Furthermore, CRS-R scores also increased markedly following treatment in both groups (both *p* < 0.001), with the experimental group scoring significantly higher than the control group (*p* < 0.001).

**Table 2:** Changes in CRS-R score, GCS score and NSE level after treatment in the experimental and control groups.

Group	CRS-R score M(*P*_25_,*P*_75_)		GCS score M(*P*_25_,*P*_75_)		NSE (ng/mL) M(*P*_25_,*P*_75_)	
	Pre-treatment	Post-treatment	Pre-treatment	Post-treatment	Pre-treatment	Post-treatment
Experimental Group	8 (6,11)	14 (14,19)	9 (7,9)	12 (11,13)	138.16 (80.00,170.83)	34.2 (23.10,50.85)
Control Group	8 (5.5,10)	12 (9.5,14)	9 (7,9)	10 (9,11)	142.63 (32.33,168.98)	63.8 (18.55,114.50)
*Z* (Experimental Group: Post-treatment vs. Pre-treatment)	6.023		6.061		6.093	
*P*	<0.001		<0.001		<0.001	
*Z* (Control Group: Post-treatment vs. Pre-treatment)	5.931		5.514		6.093	
*P*	<0.001		<0.001		<0.001	
*Z* (Post-treatment: Experimental Group vs. Control Group)	4.888		4.627		1.982	
*P*	<0.001		<0.001		0.047	

As shown in Tables 1, 3, in the experimental group, 16 patients were in a state of unresponsive wakefulness syndrome (UWS) before treatment. After treatment, 14 of these patients transitioned to a minimally conscious state (MCS), and one patient improved to emergence from MCS (EMCS). In the control group, 15 patients were in UWS before treatment, of whom 9 transitioned to MCS after treatment. Before treatment, 33 patients in the experimental group were in MCS. After treatment, 13 of these patients improved to EMCS. In the control group, 34 patients began in MCS, and only 2 transitioned to EMCS after treatment. Significantly more patients in the experimental group showed improvement in their level of consciousness compared to the control group (*p* = 0.002).

**Table 3:** Changes in state of consciousness after treatment in the experimental and control groups.

	Changes in state of consciousness (Pre-treatment→Post-treatment)					n	*χ^2^*	*P*
	UWS→UWS	UWS→MCS	UWS→EMCS	MCS→MCS	MCS→EMCS			
Experimental Group Control Group	1	14	1	20	13	49	16.494	0.002
	6	9	0	32	2	49		
n	7	23	1	52	15	98		

### 3.3 Safety

All subjects completed the scheduled treatment regimen, and no adverse events (e.g., epileptic seizures) were observed during the study.

## 4 Discussion

Adhering to the international TMS application guidelines (30, 31), this study is the first to explore the awakening-promoting effects of 5 Hz high-frequency rTMS on DOC following TBI in children. Results showed that patients in the experimental group receiving left DLPFC stimulation exhibited significantly better outcomes in serum NSE levels, GCS scores, CRS-R scores, and level of consciousness after treatment compared to the control group without rTMS treatment (*p* < 0.05). These findings suggest that 5 Hz rTMS targeting the left DLPFC can effectively promote consciousness recovery in children with DOC following TBI and reduce neurological damage.

The mechanism underlying the generation of consciousness is extraordinarily intricate (32–37) with its core lying in the brain’s effective integration and differentiation of information. This process depends on the structural intactness and functional coordination across multiple hierarchical levels, spanning from the molecular scale to the entire brain. At the structural level, the structural integrity of the cerebral cortex, thalamus, ascending reticular activating system (ARAS), brainstem, and other key components constitutes the material basis for the generation of consciousness. At the functional level, highly coordinated interactions among core brain networks—including the default mode network (DMN), central executive network (CEN), and salience network (SN)—as well as the posterior hot zone (PHZ), are critical for generating coherent, unified, and content-rich conscious experience. At the cellular and molecular level, normal excitatory-inhibitory balance, adequate energy supply, and a favorable neurochemical environment serve as the core biological substrates underpinning consciousness. TBI directly causes damage to critical brain regions in patients. The resulting diffuse axonal injury (DAI) leads to disruption of core network connectivity in the brain. Moreover, TBI triggers a series of pathological cascades (38, 39), such as an imbalance in neurotransmitters that disrupts the excitatory-inhibitory equilibrium, thereby interfering with interneuronal communication; mitochondrial dysfunction and metabolic abnormalities that compromise energy supply in brain cells; and hemodynamic changes that may result in inadequate regional cerebral blood flow, exacerbating consciousness impairment. The neurobiological mechanisms underlying the arousal-promoting effect of high-frequency rTMS may be realized through the following synergistic pathways: First, high-frequency rTMS tends to facilitate neuronal activity (40). Since the DLPFC is involved in key brain functions (e.g., working memory, decision-making, and emotion regulation) and serves as a critical node in central executive network (CEN), high-frequency rTMS targeting the DLPFC can enhance its functional connectivity with the posterior cortex (e.g., parietal lobe) (41, 42), increase local cortical excitability, and strengthen the connectivity of brain networks. Second, high-frequency rTMS promotes the expression of brain-derived neurotrophic factor (BDNF), regulates the strength of synaptic connections, and alleviates neural damage (43–47). Meanwhile, TMS modulates the balance between the excitatory neurotransmitter glutamate and the inhibitory neurotransmitter *γ*-aminobutyric acid (GABA); high-frequency rTMS typically enhances glutamate release and strengthens synaptic plasticity (48–53). Additionally, high-frequency rTMS can induce changes in local cerebral blood flow, particularly in the stimulated regions (54). Finally, high-frequency rTMS downregulates pro-inflammatory factors (e.g., TNF-*α*, IL-1β) via the let-7b-5p/HMGA2/NF-κB signaling pathway, inhibits microglial polarization toward the M1 phenotype, thereby protecting neurons and reducing neural damage (55).

The core innovation of this study lies in optimizing rTMS treatment protocols for children by accounting for the specificity of pediatric neurodevelopment. In previous adult studies, high-frequency rTMS typically employed frequencies of 10 Hz or 20 Hz, with 2,000–3,000 pulses delivered per day. However, children are not simply “miniaturized adults,” their baseline cortical excitability is higher, leading to a lower seizure threshold compared to adults (56, 57). Additionally, studies have reported that TBI enhances neuronal intrinsic excitability, significantly increasing discharge frequency and reducing threshold current (57–59). Given these factors, direct application of adult protocols may induce abnormal synchronous discharges. Although 5 Hz is lower than the 10 Hz or 20 Hz typically used in adult high-frequency rTMS treatment protocols, it still falls within the category of high-frequency stimulation and can upregulate BDNF expression, induce long-term potentiation (LTP), and enhance cortical excitability (47, 55, 60). Moreover, the 10-s inter-stimulus interval design allows for neurotransmitter replenishment, promoting cumulative rather than exhaustive pulse efficacy. For these reasons, we cautiously selected the relatively lower 5 Hz high-frequency protocol. Results confirmed that 5 Hz rTMS stimulation significantly improved consciousness levels in pediatric patients with DOC following TBI without observing severe adverse events such as seizures during treatment, preliminarily validating the safety and efficacy of this protocol in the pediatric population. This parameter choice is also indirectly supported by prior research: for instance, 3 Hz treatment protocols has been shown to be effective in adult DOC, suggesting the therapeutic potential of lower high-frequency stimulation (13).

The clinical value of this study resides in establishing a novel, safe, and effective non-invasive neuromodulation strategy for pediatric DOC following TBI. Its significance is manifested in the following key aspects: (a) It addresses an evidence gap in the field of rTMS treatment for pediatric DOC; (b) provides a critical reference for pediatric rTMS parameter optimization; and (c) Ultimately, by accelerating consciousness recovery, reducing long-term care costs, improving pediatric patients’ quality of life (QoL), and alleviating the burden on families and society, it may yield transformative impacts on clinical practice.

However, this study has certain limitations: First, the absence of long-term follow-up data after treatment completion limits the evaluation of therapeutic sustainability. Second, the assessment primarily relied on behavioral scales and serum biomarkers, with no measurements of cortical excitability or neuroplasticity biomarkers. Furthermore, the study did not incorporate neuroimaging techniques, such as functional magnetic resonance imaging (fMRI) or electroencephalography (EEG), to investigate the specific mechanisms by which rTMS modulates consciousness-related networks. Third, only a single stimulation protocol was explored, without comparison to alternative frequencies (e.g., individualized frequency), stimulation targets (e.g., PPC or M1), or combined therapeutic strategies (e.g., rTMS + tDCS). Fourth, given that TBI often alters cerebral anatomy, the sole use of the International 10–20 system for target localization—without neuronavigation—may have resulted in imprecise stimulation site placement. Finally, heterogeneity in lesion location and etiology among pediatric patients may have influenced treatment outcomes, underscoring the need for larger-sample subgroup analyses in future studies.

Based on the above findings and limitations, future research should prioritize the following directions: (a) Conducting multicenter, large-sample randomized controlled trials (RCTs) with long-term follow-up (e.g., 6–12 months) to validate the stability of treatment efficacy; (b) Detect cortical excitability indices (e.g., short-interval intracortical inhibition / long-interval intracortical facilitation, ICI/ICF) and neuroplasticity biomarkers (e.g., BDNF levels, neurofilament light chain), integrate multimodal neuroimaging (fMRI/EEG) and brain network analysis, dynamically track the effects of rTMS on the connectivity and activity of consciousness networks, and elucidate its underlying mechanisms. (c) Evaluating the synergistic effects of rTMS combined with other arousal-promoting modalities. (d) Utilize neuro-navigation technology to precisely identify stimulation sites and explore individualized stimulation parameters (frequency, target, intensity) based on individual lesion characteristics or neurofunctional status. (e) Investigating long-term neurocognitive function, motor recovery, and QoL outcomes in pediatric patients post-arousal.

## 5 Conclusion

This study first demonstrates that applying 5 Hz high-frequency rTMS targeting the left DLPFC on the basis of conventional rehabilitation can safely and effectively promote consciousness recovery in pediatric patients with DOC following TBI. This innovative protocol significantly improved patients’ GCS and CRS-R scores, accompanied by a concurrent decrease in serum NSE levels, suggesting it exerts both arousal-promoting and neuroprotective effects. Optimizing treatment parameters based on pediatric neurodevelopmental characteristics provides the first non-invasive neuromodulation strategy tailored to this population. The 5 Hz parameters optimized based on pediatric neurodevelopmental characteristics provide the first non-invasive neuromodulation strategy tailored to this population, filling a critical gap in the field. Future research should focus on large-scale long-term follow-up studies and multimodal mechanistic investigations to further validate the durability of efficacy and advance the optimization of personalized treatment protocols.

## Data Availability

The raw data supporting the conclusions of this article will be made available by the authors, without undue reservation.

## References

[1] Blackwell LS Grell R. Pediatric traumatic brain injury: impact on the developing brain. Pediatr Neurol. (2023) 148:215–22. doi: 10.1016/j.pediatrneurol.2023.06.019, PMID: 37652817

[2] Dewan MC Mummareddy N Wellons JC Bonfield CM. Epidemiology of global pediatric traumatic brain injury: qualitative review. World Neurosurg. (2016) 91:497–509. doi: 10.1016/j.wneu.2016.03.045 27018009

[3] Li Y Chen F Zhang J Li G Yang X Lu Q. Epidemiological characteristics of Chinese paediatric traumatic brain injury inpatients. Brain Inj. (2017) 31:1094–101. doi: 10.1080/02699052.2017.1298004, PMID: 28506081

[4] Liu Z Zhang X Yu B Wang J Lu X. Effectiveness on level of consciousness of non-invasive neuromodulation therapy in patients with disorders of consciousness: a systematic review and meta-analysis. Front Hum Neurosci. (2023) 24:1129254. doi: 10.3389/fnhum.2023.1129254 PMC10246452 37292582

[5] Pinto PS Poretti A Meoded A Tekes A Huisman TAGM. The unique features of traumatic brain injury in children. Review of the characteristics of the pediatric skull and brain, mechanisms of trauma, patterns of injury, complications and their imaging findings—part 1. J Neuroimaging. (2012) 22:e1–e17. doi: 10.1111/j.1552-6569.2011.00688.x, PMID: 22273264

[6] Park ES Yang HJ Park JB. Pediatric traumatic brain injury: the epidemiology in Korea. J Korean Neurosurg Soc. (2022) 65:334–41. doi: 10.3340/jkns.2021.0306, PMID: 35468704 PMC9082131

[7] Dong L Li H Dang H Zhang X Yue S Zhang H. Efficacy of non-invasive brain stimulation for disorders of consciousness: a systematic review and meta-analysis. Front Neurosci. (2023) 11:1219043. doi: 10.3389/fnins.2023.1219043 PMC10366382 37496734

[8] Yang Z Yue T Zschorlich VR Li D Wang D Qi F. Behavioral effects of repetitive transcranial magnetic stimulation in disorders of consciousness: a systematic review and Meta-analysis. Brain Sci. (2023) 13:1362. doi: 10.3390/brainsci13101362, PMID: 37891731 PMC10605911

[9] O'Neal CM Schroeder LN Wells AA Chen S Stephens TM Glenn CA. Patient outcomes in disorders of consciousness following transcranial magnetic stimulation: a systematic review and Meta-analysis of individual patient data. Front Neurol. (2021) 12:694970. doi: 10.3389/fneur.2021.694970, PMID: 34475848 PMC8407074

[10] Wan X Zhang Y Li Y Song W. An update on noninvasive neuromodulation in the treatment of patients with prolonged disorders of consciousness. CNS Neurosci Ther. (2024) 30:e14757. doi: 10.1111/cns.14757, PMID: 38747078 PMC11094579

[11] Li Y Li L Huang H. Effect of non-invasive brain stimulation on conscious disorder in patients after brain injury: a network meta-analysis. Neurol Sci. (2023) 44:2311–27. doi: 10.1007/s10072-023-06743-7, PMID: 36943589

[12] Hu Y Hu L Wang Y Luo X Zhao X He L. The effects of non-invasive brain stimulation on disorder of consciousness in patients with brain injury: a systematic review and meta-analysis of randomized controlled trial. Brain Res. (2024) 1822:148633. doi: 10.1016/j.brainres.2023.148633, PMID: 37839670

[13] Shen LB Ouyang H Yang CY Lin ZP Mou ZW Wang H. The awakening effect of high-frequency repetitive transcranial magnetic stimulation on consciousness disorder after severe craniocerebral injury. Chin J Rehabil Med. (2019) 34:1411–7.

[14] Guo S Yin Z Lu G Huang X. A comparative study of repetitive transcranial magnetic stimulation at different frequencies in arousal for coma patients after brain stem injury. Chin J Neuromed. (2019) 18:550–4. doi: 10.3760/cma.j.issn.1671-8925.2019.06.002

[15] Marangos PJ Polak JM Pearse AGE. Neuron-specific enolase. Trends Neurosci. (1982) 5:193–6. doi: 10.1016/0166-2236(82)90112-6, PMID: 6120757

[16] Bezek S Biberthaler P Martinez-Espina I Bogner-Flatz V. Pathophysiology and clinical implementation of traumatic brain injury biomarkers: neuron-specific enolase. Biomark Traum Brain Inj. (2020):169–82. doi: 10.1016/b978-0-12-816346-7.00011-7

[17] Liu F Li H Hong X Liu Y Yu Z. Research progress of neuron-specific enolase in cognitive disorder: a mini review. Front Hum Neurosci. (2024) 18. doi: 10.3389/fnhum.2024.1392519, PMID: 39040086 PMC11260780

[18] Chiaretti A Barone G Riccardi R Antonelli A Pezzotti P Genovese O. NGF, DCX, and NSE upregulation correlates with severity and outcome of head trauma in children. Neurology. (2009) 72:609–16. doi: 10.1212/01.wnl.0000342462.51073.06 19221293

[19] Tokshilykova AB Sarkulova ZN Kabdrakhmanova GB Utepkaliyeva AP Tleuova AS Satenov ZK. Neuron-specific markers and their correlation with neurological scales in patients with acute neuropathologies. (2020) 70:1267–73. doi: 10.1007/s12031-020-01536-5 32350763

[20] El-Maraghi S Yehia H Hossam H Yehia A Mowafy H. The prognostic value of neuron specific enolase in head injury. Egypt J Crit Care Med. (2013) 1:25–32. doi: 10.1016/j.ejccm.2012.12.002

[21] Menon DK Schwab K Wright DW Maas AI Demographics and Clinical Assessment Working Group of the International and Interagency Initiative toward Common Data Elements for Research on Traumatic Brain Injury and Psychological Health. Position statement: definition of traumatic brain injury. Arch Phys Med Rehabil. (2010) 91:1637–40. doi: 10.1016/j.apmr.2010.05.017, PMID: 21044706

[22] Bernat JL. Chronic consciousness disorders. Annu Rev Med. (2009) 60:381–92. doi: 10.1146/annurev.med.60.060107.091250, PMID: 19630578

[23] Ni YY Wang SH Song WQ Li BQ Chen JL Feng Z. Chinese expert consensus on neurocritical rehabilitation [J]. Chin J Rehabil Med. (2018) 33:7–14.

[24] Pan YH Sun YZ Liu XT. Advances in clinical application and mechanism research of acupuncture for disorders of consciousness. World Chin Med. (2025) 20:1052–1055+1060.

[25] Huang Z Chen Y Xiao Q Kuang W Liu K Jiang Y. Effect of acupuncture for disorders of consciousness in patients with stroke: a systematic review and meta-analysis. Front Neurol. (2022) 13:930546. doi: 10.3389/fneur.2022.930546, PMID: 36277925 PMC9581330

[26] Zhi N Sun N Huang P Yang LY Guo CX Xiong J. Acupuncture-assisted therapy for prolonged disorders of consciousness: study protocol for a randomized, conventional-controlled, assessor-and-statistician-blinded trial. Front Neurol. (2024) 15. doi: 10.3389/fneur.2024.1334483, PMID: 39291097 PMC11407111

[27] Liu Y Sun N Xiong J Zhou Y Ye X Jiang H. Modulation of cerebral cortex activity by acupuncture in patients with prolonged disorder of consciousness: an fNIRS study. Front Neurosci. (2022) 16. doi: 10.3389/fnins.2022.1043133, PMID: 36523434 PMC9744766

[28] Li XR Chen YH Li YQ Li JF Wang HY Sun W. Effect of Xingnao Kaiqiao acupuncture on arousal of consciousness disorder in children with early severe traumatic brain injury. Chin Acupunct Moxibust. (2023) 43:277–81. doi: 10.13703/j.0255-2930.20220524-k0007 36858388

[29] Wang Y t Yu J Zhuang L. Professor ZHUANG Li-xing’s experience of mind-regulation acupuncture for psychosomatic disorders. Zhongguo zhen jiu = Chinese acupuncture & moxibustion. (2023) 215–22. doi: 10.13703/j.0255-2930.20221016-k0001 37068815

[30] Rossi S Antal A Bestmann S Bikson M Brewer C Brockmöller J. Safety and recommendations for TMS use in healthy subjects and patient populations, with updates on training, ethical and regulatory issues: expert guidelines. Clin Neurophysiol. (2021) 132:269–306. doi: 10.1016/j.clinph.2020.10.003, PMID: 33243615 PMC9094636

[31] Rossi S Hallett M Rossini PM Pascual-Leone A Safety of TMS Consensus Group. Safety, ethical considerations, and application guidelines for the use of transcranial magnetic stimulation in clinical practice and research. Clin Neurophysiol. (2009) 120:2008–39. doi: 10.1016/j.clinph.2009.08.016, PMID: 19833552 PMC3260536

[32] Tononi G Koch C. Consciousness: here, there and everywhere? Philos Trans R Soc Lond Ser B Biol Sci. (2015) 370:20140167. doi: 10.1098/rstb.2014.0167, PMID: 25823865 PMC4387509

[33] Tsytsarev V. Methodological aspects of studying the mechanisms of consciousness. Behav Brain Res. (2022) 419:113684. doi: 10.1016/j.bbr.2021.113684, PMID: 34838578

[34] Seth AK Bayne T. Theories of consciousness. Nat Rev Neurosci. (2022) 23:439–52. doi: 10.1038/s41583-022-00587-4, PMID: 35505255

[35] Ihalainen R Gosseries O Van de Steen F Raimondo F Panda R Bonhomme V. How hot is the hot zone? Computational modelling clarifies the role of parietal and frontoparietal connectivity during anaesthetic-induced loss of consciousness. bioRxiv. (2020). doi: 10.1101/2020.12.19.423595 33577934

[36] Liyana Arachige M Seneviratne U John N Ma H Phan TG. Mapping topography and network of brain injury in patients with disorders of consciousness. Front Neurol. (2023) 29:1027160. doi: 10.3389/fneur.2023.1027160 PMC10090673 37064187

[37] Koyya P Manthari RK Pandrangi SL. Brain-derived neurotrophic factor—the protective agent against neurological disorders. CNS Neurol Disord Drug Targets. (2024) 23:353–66. doi: 10.2174/1871527322666230607110617, PMID: 37287291 PMC11348470

[38] Giacino JT Fins JJ Laureys S Schiff ND. Disorders of consciousness after acquired brain injury: the state of the science. Nat Rev Neurol. (2014) 10:99–114. doi: 10.1038/nrneurol.2013.279, PMID: 24468878

[39] Panda R Thibaut A Lopez-Gonzalez A Escrichs A Bahri MA Hillebrand A. Disruption in structural–functional network repertoire and time-resolved subcortical fronto-temporoparietal connectivity in disorders of consciousness. eLife. (2022) 11. doi: 10.7554/eLife.77462, PMID: 35916363 PMC9385205

[40] Klomjai W Katz R Lackmy-Vallée A. Basic principles of transcranial magnetic stimulation (TMS) and repetitive TMS (rTMS). Ann Phys Rehabil Med. (2015) 58:208–13. doi: 10.1016/j.rehab.2015.05.005, PMID: 26319963

[41] Tik M Hoffmann A Sladky R Tomova L Hummer A Navarro de Lara L. Towards understanding rTMS mechanism of action: stimulation of the DLPFC causes network-specific increase in functional connectivity. NeuroImage. (2017) 15:289–96. doi: 10.1016/j.neuroimage.2017.09.022, PMID: 28912081

[42] Veniero D Maioli C Miniussi C. Potentiation of short-latency cortical responses by high-frequency repetitive transcranial magnetic stimulation. J Neurophysiol. (2010) 104:1578–88. doi: 10.1152/jn.00172.2010, PMID: 20631218

[43] Luo J Zheng H Zhang L Zhang Q Li L Pei Z. High-frequency repetitive transcranial magnetic stimulation (rTMS) improves functional recovery by enhancing neurogenesis and activating BDNF/TrkB signaling in ischemic rats. Int J Mol Sci. (2017) 18:455. doi: 10.3390/ijms18020455, PMID: 28230741 PMC5343989

[44] Pan F Mou T Shao J Chen H Tao S Wang L. Effects of neuronavigation-guided rTMS on serum BDNF, TrkB and VGF levels in depressive patients with suicidal ideation. J Affect Disord. (2023) 15:617–23. doi: 10.1016/j.jad.2022.11.059 36462609

[45] Li H Shang J Zhang C Lu R Chen J Zhou X. Repetitive transcranial magnetic stimulation alleviates neurological deficits after cerebral ischemia through interaction between RACK1 and BDNF exon IV by the phosphorylation-dependent factor MeCP2. Neurotherapeutics. (2020) 17:651–63. doi: 10.1007/s13311-019-00771-y, PMID: 31912469 PMC7283432

[46] Ma Q Geng Y Wang HL Han B Wang YY Li XL. High frequency repetitive transcranial magnetic stimulation alleviates cognitive impairment and modulates hippocampal synaptic structural plasticity in aged mice. Front Aging Neurosci. (2019) 11:235. doi: 10.3389/fnagi.2019.00235, PMID: 31619982 PMC6759649

[47] Shang Y Wang X Shang X Zhang H Liu Z Yin T. Repetitive transcranial magnetic stimulation effectively facilitates spatial cognition and synaptic plasticity associated with increasing the levels of BDNF and synaptic proteins in Wistar rats. Neurobiol Learn Mem. (2016) 134 Pt B:369-78) 134:369–78. doi: 10.1016/j.nlm.2016.08.016, PMID: 27555233

[48] Sharbafshaaer M Cirillo G Esposito F Tedeschi G Trojsi F. Harnessing brain plasticity: the therapeutic power of repetitive transcranial magnetic stimulation (rTMS) and Theta burst stimulation (TBS) in neurotransmitter modulation, receptor dynamics, and neuroimaging for neurological innovations. Biomedicine. (2024) 12:2506. doi: 10.3390/biomedicines12112506, PMID: 39595072 PMC11592033

[49] Jannati A Oberman LM Rotenberg A Pascual-Leone A. Assessing the mechanisms of brain plasticity by transcranial magnetic stimulation. Neuropsychopharmacology. (2023) 48:191–208. doi: 10.1038/s41386-022-01453-8, PMID: 36198876 PMC9700722

[50] Bagnato S Boccagni C Sant'Angelo A Prestandrea C Rizzo S Galardi G. Patients in a vegetative state following traumatic brain injury display a reduced intracortical modulation. Clin Neurophysiol. (2012) 123:1937–41. doi: 10.1016/j.clinph.2012.03.014, PMID: 22560638

[51] Yue L Xiao-lin H Tao S. The effects of chronic repetitive transcranial magnetic stimulation on glutamate and gamma-aminobutyric acid in rat brain. Brain Res. (2009) 1260:94–9. doi: 10.1016/j.brainres.2009.01.009, PMID: 19401169

[52] Wang J Ding C Fu R Zhang Z Zhao J Zhu H. Effect of repeated transcranial magnetic stimulation on excitability of glutaminergic neurons and gamma-aminobutyric neurons in mouse hippocampus. Sheng Wu Yi Xue Gong Cheng Xue Za Zhi. (2025) 42:73–81. doi: 10.7507/1001-5515.202405025, PMID: 40000178 PMC11955338

[53] Surendrakumar S Rabelo TK Campos ACP Mollica A Abrahao A Lipsman N. Neuromodulation therapies in pre-clinical models of traumatic brain injury: systematic review and translational applications. J Neurotrauma. (2023) 40:435–48. doi: 10.1089/neu.2022.0286, PMID: 35983592

[54] Shang YQ Xie J Peng W Zhang J Chang D Wang Z. Network-wise cerebral blood flow redistribution after 20 Hz rTMS on left dorso-lateral prefrontal cortex. Eur J Radiol. (2018) 101:144–8. doi: 10.1016/j.ejrad.2018.02.018, PMID: 29571788

[55] Hameed MQ Dhamne SC Gersner R Kaye HL Oberman LM Pascual-Leone A. Transcranial magnetic and direct current stimulation in children. Curr Neurol Neurosci Rep. (2017) 17:11. doi: 10.1007/s11910-017-0719-0, PMID: 28229395 PMC5962296

[56] Määttä S Könönen M Kallioniemi E Lakka T Lintu N Lindi V. Development of cortical motor circuits between childhood and adulthood: a navigated TMS-HdEEG study. Hum Brain Mapp. (2017) 38:2599–615. doi: 10.1002/hbm.23545, PMID: 28218489 PMC6866783

[57] Nichols J Perez R Wu C Adelson PD Anderson T. Traumatic brain injury induces rapid enhancement of cortical excitability in juvenile rats. CNS Neurosci Ther. (2015) 21:193–203. doi: 10.1111/cns.12351, PMID: 25475223 PMC5880220

[58] Seeger TA Kirton A Esser MJ Gallagher C Dunn J Zewdie E. Cortical excitability after pediatric mild traumatic brain injury. Brain Stimul. (2017) 10:305–14. doi: 10.1016/j.brs.2016.11.011, PMID: 27916406

[59] Karimi SA Hosseinmardi N Sayyah M Hajisoltani R Janahmadi M. Enhancement of intrinsic neuronal excitability-mediated by a reduction in hyperpolarization-activated cation current (Ih) in hippocampal CA1 neurons in a rat model of traumatic brain injury. Hippocampus. (2021) 31:156–69. doi: 10.1002/hipo.23270, PMID: 33107111

[60] Moisello C Blanco D Fontanesi C Lin J Biagioni M Kumar P. TMS enhances retention of a motor skill in Parkinson's disease. Brain Stimul. (2015) 8:224–30. doi: 10.1016/j.brs.2014.11.005, PMID: 25533243 PMC4314317

